# Mild Traumatic Brain Injury: Lessons Learned from Clinical, Sports, and Combat Concussions

**DOI:** 10.1155/2012/371970

**Published:** 2012-03-27

**Authors:** Judy C. Kelly, Efland H. Amerson, Jeffrey T. Barth

**Affiliations:** Department of Psychiatry & Neurobehavioral Sciences, University of Virginia, Charlottesville, VA 22908, USA

## Abstract

Over the past forty years, a tremendous amount of information has been gained on the mechanisms and consequences of mild traumatic brain injuries. Using sports as a laboratory to study this phenomenon, a natural recovery curve emerged, along with standards for managing concussions and returning athletes back to play. Although advances have been made in this area, investigation into recovery and return to play continues. With the increase in combat-related traumatic brain injuries in the military setting, lessons learned from sports concussion research are being applied by the Department of Defense to the assessment of blast concussions and return to duty decision making. Concussion management and treatment for military personnel can be complicated by additional combat related stressors not present in the civilian environment. Cognitive behavioral therapy is one of the interventions that has been successful in treating symptoms of postconcussion syndrome. While we are beginning to have an understanding of the impact of multiple concussions and subconcussive blows in the sports world, much is still unknown about the impact of multiple blast injuries.

## 1. Introduction

As early as 1962, Symonds made the observation that any concussion, no matter how mild, may result in permanent neurologic impairment [1, 2]. Almost a decade later, researchers in New Zealand first documented delayed return to work and complex processing speed deficits related to “minor head injury” [3]. By the early 1980's, neuroscientists at the University of Virginia were finding that patients who had sustained a concussion were slow to return to work and were continuing to experience deficits in attention, memory, new problem solving, mental flexibility, and cognitive processing speed three months postinjuries [4, 5].

Around this same time, animal studies were revealing neuropathological/histological changes in the brain stems of primates when subjected to acceleration deceleration, rotational injuries, or mild traumatic brain injuries (mTBIs) [2, 6]. These acceleration or torque forces were expressed as “shear-strain”, or stretching and tearing of axonal fibers. Concurrent neurochemical cascades occur during any severity of traumatic brain injury (TBI). These reactions to trauma typically involve the rapid transfer of potassium and sodium to the extracellular space, the influx of calcium, and the release of oxygen free radicals, which can result in cell death (apoptosis). Returning to a homeostatic state requires energy (glucose) and time, which in the case of mild concussion is often 5 to 10 days.

To better understand the neurocognitive sequel of this concussion phenomenon in humans, a new method of study was initiated at the University of Virginia: the Sports as a Laboratory Assessment Model (SLAM) [2]. This research model involved the use of contact sports as a controlled laboratory to collect brief baseline neurocognitive test data that would later be compared to repeat postconcussion test data. In the first study of its kind, more than 2300 football players at 10 universities were assessed before the football season with brief neurocognitive tests [7]. When a concussion occurred in these players, the same preseason battery was administered 24 hours, 5 days, and 10 days postinjury. Scores on these measures were not only compared to the preseason performance of the individual athlete, but also to a matched control (redshirt) group (a student athlete who delays eligibility to play, typically during their freshman year of college) at the same time intervals. On neurocognitive tests, significant differences were noted between the concussed players' baseline test scores and the concussed and control players' test scores during the 24 hour and 5 day assessments. By the 10 day reevaluation, players who had sustained a concussion were performing at nearly the same level as their matched controlled peers. This model suggested that there is a typical, and relatively quick, recovery curve that occurred in healthy, young, motivated athletes when they were allowed to rest. This is especially true when there are no complicating psychosocial or medical factors, which are more often seen in clinical populations.

The SLAM model was initially instituted as a controlled laboratory to more accurately determine the acute neurocognitive cost to any individual suffering a single mild concussion, and to plot the typical recovery curve for clinical applications. Today this model is a standard part of the sports medicine concussion assessment and management programs at many high schools, universities, and professional sports teams, to protect athletes from the dangers of returning to play before complete resolution of neurologic and cognitive concussion symptoms. By having a preseason baseline assessment, athletic trainers and team physicians can better evaluate and treat concussions when they occur. This helps to ensure that an injury, no matter how mild, can be properly assessed and treated appropriately in order to reduce the potential severe consequences of a second injury. A rare condition known as second impact syndrome (SIS) [8] can occur, especially in adolescents who have sustained two minor impacts in close proximity. It is hypothesized that these closely occurring injuries can result in a catastrophic increase in intracranial pressure due to dysfunction of autoregulation of the cerebrovascular system. Identifying concussions in order to allow full neurologic recovery is critical to avoid further injury and to facilitate prudent return-to-play (RTP) decisions.

## 2. Return to Play

Based on this line of research which has been expanded and refined by many other neuroscientists, specific recommendations have been created to guide safe return-to-play (RTP) decisions. In 2008, the Third International Conference on Concussion in Sport was held in Zurich which produced a consensus on managing sports concussions [9]. Based on these guidelines, a gradual RTP protocol was created. The protocol goes into effect once an athlete is symptom free while at rest. As the athlete completes each component and remains symptom free, he or she moves to the next step. First, light aerobic exercise, which keeps the heart rate at less than 70% of the maximum, predicted heart rate is recommended. If symptoms do not return, the second stage involves engaging in sport-specific exercises that do not involve any potential impact to the head. For example, these would be skating in ice hockey or running in soccer. Third, noncontact training drills are added, such as passing drills in ice hockey or football, along with beginning progressive resistance training. Fourth, the athlete can engage in a full contact practice and return to normal training activities. Once they have reached this stage without experiencing a return of symptoms, they are able to return to full play.

## 3. Application of SLAM to Military Concussions

Another area where concussions have gained significant attention is in the current conflicts in Iraq and Afghanistan. One of the signature injuries of Operation Iraqi Freedom (OIF), Operation Enduring Freedom (OEF), and now Operation New Dawn (OND) has been traumatic brain injury. Approximately 79% of combat-related TBIs are caused by improvised explosive devices, leading to blast concussive injuries [10]. Traumatic brain injuries that occur as a result of a blast can involve any aspect of the four blast phases [11].

The primary phase is characterized by atmospheric overpressurization immediately followed by a vacuum or atmospheric underpressurization as a function of the initial blast wave. Typical injuries resulting from this blast phase are damage to hollow organs such as rupture of the tympanic membranes, pulmonary barotrauma, and acute gas emboli (AGE) in the cerebrovasculature and cerebrospinal fluid [12, 13]. The secondary phase involves objects that are placed in motion due to the blast leading to blunt and penetrating injuries [12, 13]. For example, a secondary phase of injury can occur when blast debris (rocks, cement, etc.) hits a service member. The tertiary blast phase occurs when service members are thrown off of their feet and hit stationary objects, which often results in acceleration-deceleration and blunt head trauma injuries. Quaternary-phase injuries are sustained following a blast and may include burns, toxic inhalation, exposure to radiation, asphyxiation, dust inhalation, and crush trauma [12, 13]. The primary and quaternary phases of combat blast injury make it considerably more complex than the typical sports related concussion, which involves blunt trauma and acceleration-deceleration dynamics (similar to secondary and tertiary phases in combat blast).

## 4. Using SLAM in the Military: Return to Duty

The SLAM baseline methodology was recently engaged by the military to better assess and manage concussions. Concussions are identified in the field by a combination of description of blast exposure (being within 50 meters of a blast), symptom report, and use of a brief mental status/neurocognitive assessment measure, the Military Acute Concussion Evaluation (MACE) [14], which is based upon the standardized Assessment of Concussion (SAC) from the sports literature [15]. In May 2008, the military began using the Automated Neuropsychological Assessment Metric (ANAM) to assess neurocognitive functioning in all predeployers. This provided baseline neurocognitive data for military members to compare to postconcussive test results to determine the extent of injury.

 Using the sports concussion literature, the military has created guidelines for returning military members to duty following a concussion. In 2010, a statement was issued that required designated periods of rest following a concussion (Deputy Secretary of Defense). Procedures were established that specify when a concussion evaluation is mandatory. Clinical practice guidelines are available to medical personnel in the deployed environment regarding the evaluation and treatment of concussions. These mandatory events are tracked and reported to Department of Defense Executive Agent's Joint Trauma analysis and Prevention of Injury in Combat (JTAPIC) program office. Additionally, there are currently specific teams in the deployed environment, Concussion Care Centers that assess and treat traumatic brain injuries.

## 5. Treatment in CONUS

 Once a TBI occurs, rehabilitation begins, whether on the battlefield or in the Continental United States (CONUS). The symptoms of most mild TBIs will resolve with rest shortly after the injury (typically 48 hours to 3 months). However, if symptoms do not resolve with rest in 10–14 days or if the service member has had multiple concussions, they are evacuated to a higher level of in-theater care [16] to be evaluated by a specialized TBI medical unit. If their symptoms are severe, they can be med-evaced out of theater and admitted to an Acute Care Unit at a military treatment facility (MTF) for medical stabilization and to begin undergoing assessments by psychiatry, neurology, neuropsychology, physical therapy, occupational therapy, and speech and language pathology [17].

 As patients improve, they transition to acute inpatient rehabilitation [17] with a goal to “set the stage for optimal community reintegration post discharge and to restore functional independence” (Page 185). Many individuals with TBIs will make effective recoveries during acute rehabilitation programs. Those that do not may be referred to transitional rehabilitation programs with a goal of community reintegration or a return to the least restrictive environment. Transitional rehabilitation programs may be residential, community-based day treatment as part of a larger medical system, or a home-based model.

 The U.S. Department of Veterans Affairs has designed a polytrauma system of care (PSC) to offer specialized rehabilitation to veterans in areas closer to their home than most major military treatment centers [17]. There are four polytrauma rehabilitation centers (PRCs), spread across the USA (Minnesota, California, Virginia, and Florida) with a goal of acting as a hub for acute medical and rehabilitation care, along with research and education in the fields of polytrauma and TBI.

 The Defense Centers of Excellence (DCoE) houses the Defense and Veterans Brain Injury Center (DVBIC). DVBIC brings together the DoD, VA, and civilian providers to assess and treat TBIs, provide education, and conduct clinical research. In addition to active duty military members and veterans, DVBIC also provides care to military beneficiaries who have sustained a TBI. DVBIC is comprised of 19 centers including MTFs, VAMCs, and two civilian TBI community reentry partners spread across the USA and in Germany.

## 6. Traumatic Brain Injury and Posttraumatic Stress Disorder

One of the complicating factors in recovery from concussion that is seen in the military population that is typically not seen in athletes is that of psychosocial stressors. These include sleep deprivation or alternated sleep schedules and mental health concerns, including acute stress disorder and at times posttraumatic stress disorder. When working with returning veterans, it is important for providers to be able to determine whether an individual has a mild TBI that has not resolved which is referred to as Postconcussion Syndrome (PCS)/Postconcussion Disorder (PCD), Posttraumatic Stress Disorder (PTSD)/posttraumatic spectrum disorder, or both. While symptom management is key, knowing the root cause of a symptom such as irritability, can direct treatment that targets the root cause and improves the efficiency of the recovery process. While most mild TBI symptoms resolve within 1–3 months of the event, in rare cases symptoms may persist longer. When the neuropsychological symptoms last beyond the initial 1–3 month time period, PCS is diagnosed. Common symptoms of PCS are difficulty with memory, changes in mood, problems paying attention and concentrating, decreased energy, and mental slowness. Despite advances in our understanding of mTBI, it is still unclear why some individuals recover faster than others after a mild TBI. Undoubtedly, recovery is a multifactorial process based upon age, gender, genetic predisposition, premorbid neurocognitive abilities, severity of injury, number of previous concussions, substance abuse history, sleep disturbances, pain, depression, and psychosocial supports to name a few.

The sequelae of an mTBI are varied and have far reaching implications for the individual if not clinically addressed. Feelings of loss and reduced functioning may lead to depression, in conjunction with having ecological impacts, such as relationship issues and decreased work performance. Untreated depression and other psychiatric conditions that can be comorbid with mTBI not only impede psychosocial functioning but also reduce the amenability to treatment [18]. The increased propensity for an individual to experience psychiatric symptoms with mTBI requires treatment methodologies which address both the neurologic and emotional injury. Because of the variability of neurologic and emotional injury manifestations, standard approaches to treatment have proven ineffective in many cases.

As seen with treating depression, cognitive behavioral therapy (CBT) has also been helpful in ameliorating anxiety spectrum disorders such as Obsessive Compulsive (OCD) and Posttraumatic Stress Disorders (PTSD). Williams and Evans [19] postulated that anxiety spectrum disorders can be just as debilitating as depression and if left untreated often result in reduced quality of life and maladaptive coping mechanisms. Until recently, there has been a scarcity of research evaluating the treatment of depression and other psychiatric disorders related to mTBI [18]. The first line of therapy to help remediate depression and anxiety symptoms during the course of neurorehabilitation has been behavioral therapy (BT) [18]. As a result of an mTBI, often maladaptive behaviors emerge in those who have prolonged impairments. Schlund and Pace [20] suggest that BT has been effective enhancing social skills and cognitive rehabilitation.

Treatment protocols for CBT have been modified to apply to those less capable of participating in traditional CBT approaches for psychiatric conditions [19]. More often than not, emotional and cognitive symptoms persist after mTBI and are maintained by dysfunctional thought processes and emotional impairment. Early research suggests addressing these emotional issues in the initial stages of treatment, simultaneously with the neurologic injury has been helpful in preventing postconcussion sequelae [21].

Tiersky and colleagues [22] noted that with a combination of cognitive remediation and CBT, participants reported less emotional distress and increased cognitive functioning after treatment. Additionally, the emotional improvements continued to increase at the 1-month and 3-month followups posttreatment. The cognitive remediation portion of the treatment focused on attention, information processing, and memory. The focus of CBT was to provide increased coping behaviors, teach skills to prevent relapse, and cope with the feelings of loss related to their decreased functioning. Although the treatment was not purely CBT, it provides additional evidence that CBT can be effective with patients who have sustained both mild and moderate TBIs.

A more recent study of an individualized treatment of CBT was also found effective in reducing emotional distress in patients with a TBI [23]. In this research, CBT was modified from its standard form to include repetition of important concepts and frequent breaks. Because cognitive assessments were completed prior to the study, the intervention was individualized to each patient based on their ability to learn and retain information and their cognitive processing speed. The intervention was delivered in both a group format and over the telephone. Those in the treatment groups had statistically significant less emotional distress following the study and at the 1-month followup. This suggests that patients who have sustained a TBI can benefit from CBT, even though some alterations may need to be made to the basic CBT concepts.

Insomnia is also commonly associated with mTBI and often prolongs the recovery process. Ouellet and Morin [24] conducted a case study of a man who sustained a brain injury and had failed pharmacotherapy for insomnia. Following eight weeks of treatment focusing on stimulus control, sleep restriction, cognitive therapy, and sleep hygiene education, the participant's sleep had improved. At the 3-month followup, the positive effects were still apparent. This case study showed that the use of CBT is promising for post-TBI insomnia. In 2007, Ouellet and Morin [25] expanded on their work. With a small group of participants (*n* = 11) who had sustained a TBI, they provided the same treatment as above and added a fatigue management component. Sleep efficiency had improved for each participant in the study at the end of treatment and the positive effects remained at the 3-month followup. This appears to be an effective way of managing insomnia in individuals with a TBI, however, more research with larger sample sizes continues to be needed in the field.

## 7. The Impact of Multiple Concussions

 While some studies have shown little to no impact of 2 concussions [26], other research has shown that multiple concussions and subconcussive blows may lead to chronic traumatic encephalopathy (CTE) [27]. Chronic traumatic encephalopathy has been found on autopsy in boxers, football players, and hockey players who suffered multiple concussions, and many more subconcussive blows, eventually exhibiting early dementia and depression. Histologically, CTE is characterized by the expression of high concentrations of tau protein (neurofibrillary tangles) and Beta Amyloid plaques. These findings appear to be relevant to the military due to the high risk of multiple blast injuries. This offers further support for the importance of new guidelines for concussion identification, management, and long-term follow-up.

## 8. Conclusion

 Almost four decades ago, mild head injury in clinical populations began to gain attention from the medical and neuropsychological communities. Much of the initial controlled data on mTBI and concussions was gleaned from athletes who were at high risk for mild head trauma. With the multiple combat tours and the nature of warfare in Iraq and Afghanistan that are putting service members at risk for multiple concussive injuries, the lessons learned from the sports concussion literature are being applied to the military. While there are differences in the mechanism of injury and additional challenges when TBIs occur in the combat setting, there are also similarities. Due to these similarities and the need for a graduated return to duty (or play), the military is now implementing clinical practice guidelines similar to the Zurich recommendations used in many civilian sports. Following a TBI, military personnel and veterans have services available to them though military medical treatment facilities and the Department of Veterans Affairs for continued treatment of symptoms.

There is continuing research on the impact of multiple concussions and subconcussive blows in boxing, football, soccer, and hockey that have led to a better understanding of the possible links to CTE. There are, however, no longitudinal bodies of data currently available from which to draw conclusions regarding the impact of multiple concussions during combat. Continued study of combat related blast concussions is important to our efforts to quickly and safely return service members to duty, treat persistent concussion symptoms, and avoid long-term sequel and CTE.
